# Progressive hearing loss in vitamin A-deficient mice which may be protected by the activation of cochlear melanocyte

**DOI:** 10.1038/s41598-018-34653-8

**Published:** 2018-11-06

**Authors:** Mia Gi, Dae Bo Shim, Ling Wu, Jinwoong Bok, Mee Hyun Song, Jae Young Choi

**Affiliations:** 1 Brain Korea 21 PLUS Project for Medical Science, Yonsei University College of Medicine, Seoul, South Korea; 2 Department of Otorhinolaryngology, Myongji Hospital, Hanyang University College of Medicine, Goyang, South Korea; 3 Department of Otorhinolaryngology, Myongji Hospital, Hanyang University Medical Center, Goyang, South Korea; 4 Department of Otorhinolaryngology, Yonsei University College of Medicine, Seoul, South Korea; 5 Department of Anatomy, Brain Korea 21 PLUS Project for Medical Science, Yonsei University College of Medicine, Seoul, South Korea; 6 Research Center for Natural Human Defense System, Yonsei University College of Medicine, Seoul, South Korea; 7 The Airway Mucus Institute, Yonsei University College of Medicine, Seoul, South Korea

## Abstract

Vitamin A deficiency (VAD) produces various pathologic phenotypes in humans and animals. However, evidence regarding the effect of VAD on hearing function has been inconsistent. In this study, we evaluated the effect of VAD on hearing function in two mouse models of VAD. Hearing ability was evaluated on the basis of auditory brainstem response from 3 to 20 weeks after birth in C57BL/6 (pigmented) and imprinting control region (albino) mice. The two mice strains were divided into the VAD (purified vitamin A-free diet from 7 days after pregnancy) and control (normal diet) groups. Albino VAD mice exhibited hearing loss after 6 weeks and became deaf at 18 weeks. Histological findings revealed degenerative changes in outer hair cells and neuronal loss in the spiral ganglion in albino VAD mice. In contrast, pigmented VAD mice, except those with middle-ear infection, showed no significant hearing loss. Interestingly, pigmented VAD mice exhibited melanocyte activation in the stria vascularis and upregulation of tyrosinase. Recovery of hearing after noise exposure was poorer in pigmented VAD mice than in control mice. In conclusion, complete VAD might be related to age-related or noise-induced hearing loss in mice, protection against which might involve melanocyte activation.

## Introduction

Sensorineural hearing loss (SNHL) is one of the most common hearing problems, which affects more than 30% of the individuals over 65 years of age. It has several known risk factors, such as noise exposure, aging, and ototoxic drugs, but the degree of hearing loss they cause varies among individuals. Furthermore, the mechanism underlying the effect of these risk factors on hearing loss remains unclear.

Vitamin A is essential for a variety of physiological process, including vision, skin differentiation, spermatogenesis, and immune response^1,2^. Previous epidemiological studies have suggested that vitamin A deficiency (VAD) may be associated with hearing loss^3–5^. Michikawa *et al*. found that increased serum levels of retinol and provitamin A (carotenoids) were associated with the decreased prevalence of hearing impairment among the Japanese^6^. VAD is also known to increase noise susceptibility in guinea pigs^7^. However, the direct effect of VAD on hearing function remains controversial. A previous study reported no VAD-induced morphological changes in the cochlea of guinea pigs^8^, and another showed that vitamin A did not play an important role in the development, metabolism, and function of the rat cochlea^9^. Moreover, other studies have reported the presence of binding sites for vitamin A in the stria vascularis and high vitamin A levels in the whole cochlea of guinea pigs^10^. These discrepant findings regarding the role of vitamin A in cochlear function appear to stem from genetic differences even within the same species, as well as differences in the methodology used for establishing VAD mouse models. In particular, one of the major limitations of previous studies was that VAD models were created by placing animals on a VAD diet from 2 to 3 weeks after birth, by which time, the plasma retinol concentrations could be maintained within a considerable range by the mobilisation of hepatic retinol reserves^7,9,11^.

Melanocytes present in the cochlea have an essential function in inner ear physiology. They protect against various types of hearing loss, including age-related hearing loss (ARHL) and noise-related hearing loss (NIHL), by means of calcium buffering, heavy metal scavenging, and antioxidant activities^12,13^. Previous studies have reported that melanocyte deficiency results in various hearing impairments in mammals^14,15^.

Therefore, we established complete VAD in pigmented and albino mouse models to evaluate the effect of VAD on hearing loss.

## Results

### Body weight and general morbidity in VAD mice

**Figure 1:**
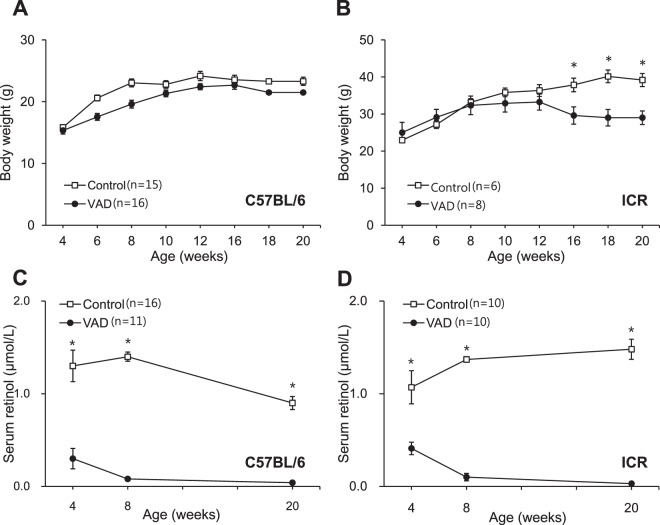
Body weights and serum retinol concentrations of the vitamin A-deficient (VAD) mice. Body weights of control and VAD mice of the pigmented (**A**) and albino (**B**) types. Serum retinol concentrations of the control and VAD mice of the pigmented (**C**) and albino (**D**) types at 4, 8, and 20 weeks of age. Values are expressed as mean ± standard error of the mean. *P < 0.01.

While no significant differences were observed in body weight between the VAD and control groups of C57BL/6 mice until 20 weeks of age (Fig. 1A), the body weights of the VAD albino mice (imprinting control region; ICR) at 16, 18, and 20 weeks of age were significantly lower than those of the control albino mice (P < 0.05 at 16 weeks; P < 0.01 at 18 and 20 weeks; Fig. 1B). Autopsy findings revealed the liver as being yellowish and slightly enlarged in some of the 18-week-old VAD albino mice, suggesting that weight loss in the VAD group was caused by decreased immune system function in the intestine and liver after 14 weeks^16^. Although neither strain of VAD mice exhibited general morbidity by 14 weeks of age, some of the C57BL/6 mice exhibited middle-ear infection after 14 weeks. We excluded data from mice with infected ears.

### Serum retinol concentrations in VAD mice

At 4 weeks of age, the serum retinol concentrations in the VAD pigmented and albino mice (0.28 ± 0.11 and 0.41 ± 0.07 μmol/L, respectively) were significantly lower than those in the control mice (P < 0.05). While the serum retinol concentrations in the VAD pigmented and albino mice further decreased to 0.08 ± 0.03 and 0.10 ± 0.04 μmol/L, respectively, at 8 weeks of age and almost fell below the detection limit (0.03 μmol/L) at 20 weeks of age, the corresponding values in both strains of control mice remained relatively stable, ranging from 0.75 to 1.21 μmol/L (pigmented mice; Fig. 1C) and 1.07 to 1.48 μmol/L (albino mice; Fig. 1D) throughout the 20-week study period (P < 0.001).

### Changes in hearing threshold with age

**Figure 2:**
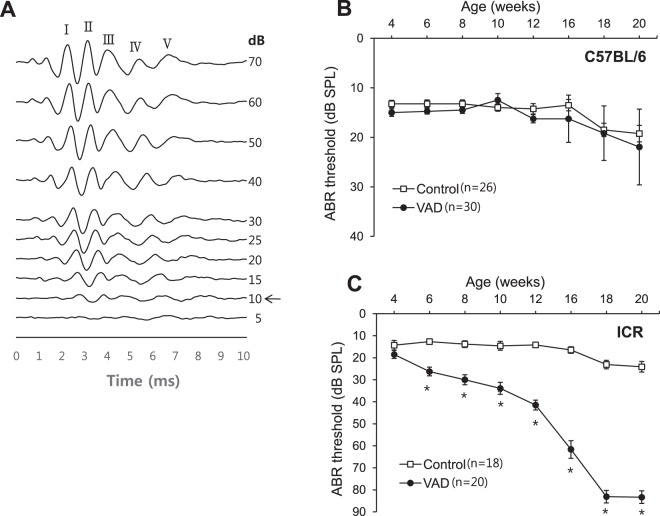
Changes in hearing threshold in response to the clicking sound with age. (**A**) Shows a sample of ABR waveforms from C57BL/6 control (4-week-old) mice. “I–V” indicates the location of ABR peaks. The black arrow indicates the ABR threshold. (**B**) In pigmented mice, no differences are observed in hearing between the control (n = 26) and VAD (n = 30) groups, except at 16 and 20 weeks of age, when two to three VAD mice developed otitis media. (**C**) The vitamin A-deficient (VAD, n = 20) albino mice exhibit a decline in hearing with age after 6 weeks and premature deafness (>80 dB hearing loss) after 18 weeks of age. *P < 0.01. Number of individuals analysed per group: VAD, VAD pigmented, and control pigmented mice.

The patterns of change in hearing threshold with age in response to the clicking sound differed substantially between the two mice strains. The VAD pigmented mice exhibited no significant changes in hearing level at 20 weeks of age, except at 14–16 weeks of age, when two mice developed middle-ear infection, which was identified using a 2.7-mm endoscope (Fig. 2B). In contrast, the VAD albino mice exhibited hearing loss at 6 weeks of age, following which, their hearing thresholds rapidly increased until 12 weeks of age, eventually attaining levels that indicated deafness (>80 dB HL) at 18 weeks of age (P < 0.001; Fig. 2C).

### Morphology of hair-cell stereociliain albino mice

**Figure 3:**
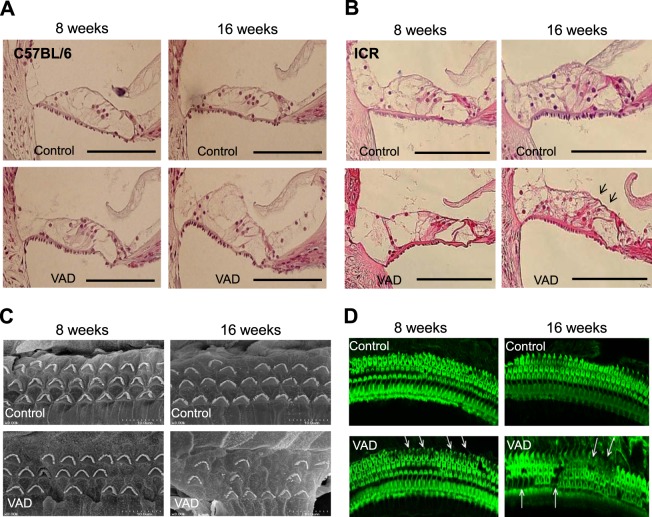
Morphology of hair-cell stereocilia in the vitamin A-deficient (VAD) pigmented mice. Representative histological findings of the organ of Corti in the pigmented (**A**, n = 8) and albino (**B**, n = 8) mice. Haematoxylin–eosin staining demonstrates outer hair cell degeneration (arrows) after 16 weeks of a VAD diet. Bars indicate 100 μm. Tissues were harvested from the middle cochlear turn of four mice from each group. (**C**) Scanning electron microscopic images of hair cell stereocilia. In contrast to the well-organised outer hair cells in the control group (n = 6), albino mice (n = 6) in the VAD group exhibit damaged outer hair cells (arrows) in the middle cochlear turn at 8 weeks of age. These degenerative changes are more obvious at 16 weeks of age. Tissues were harvested from the middle cochlear turn of three ICR mice from each group. (**D**) Confocal microscopic images (600×) of fluoresce in isothiocyanate–phalloidin stained cochlea in VAD albino mice (n = 6). The cochlear middle turn of the VAD mice exhibit some unstained areas with loss of stereocilia at 8 weeks of age; the severity of loss of stereocilia is greater among older mice (16 weeks). In contrast, the control mice exhibit three intact rows of outer hair cells. White arrows indicate the loss of hair cells. Tissues were harvested from the middle cochlear turn of three ICR mice from each group.

Analysis of haematoxylin–eosin-stained sections of the cochleae of the VAD pigmented and control mice revealed normal morphology of the organ of Corti (Fig. 3A). In contrast, the older VAD albino mice (16 weeks of age) exhibited outer hair cell damage in the middle cochlear turn (Fig. 3B).

Ultrastructural findings of stereociliain the VAD albino mice that exhibited hearing loss revealed degenerative outer hair cells in the middle cochlear turn at 8 weeks of age; this phenomenon became more apparent at 16 weeks of age. In contrast, the control mice exhibited well-preserved outer hair cells (Fig. 3C).

The findings of fluorescence microscopy performed at 8 weeks of age also revealed unstained areas in the cochlear middle turn of the VAD albino mice, indicating partial damage to the outer hair cells; these findings were in contrast to the normal stereocilia, showing greenish fluorescence, observed in the similarly aged control mice. At 16 weeks of age, the VAD albino mice exhibited more severe stereociliary damage than did the control mice (Fig. 3D).

**Figure 4:**
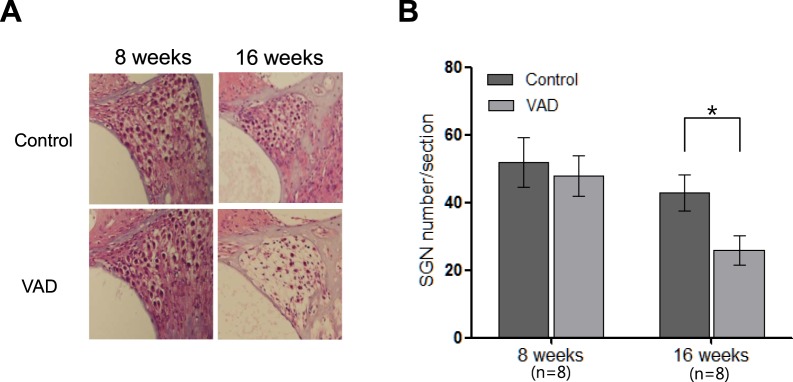
Histological findings (light microscopy images; 400×) of the spiral ganglion in control and vitamin A-deficient (VAD) albino mice. (**A**) VAD mice (n = 8) exhibit a relatively high loss of neurons in the middle cochlear turn (arrows) compared with the control mice (n = 8) at the age of 16 weeks (**B**) Summary data collected from eight mice in the middle turn. *Indicate p < 0.05 compared to control.

In addition, the VAD albino mice exhibited a greater extent of neuronal loss in the spiral ganglion (43 ± 5.4 per section) at 16 weeks of age than did the control mice (26 ± 4.3 per section, p < 0.05; Fig. 4A). These findings suggested the involvement of multiple defects in the age-dependent increase in hearing threshold.

### Increased melanosome production in VAD pigmented mice

**Figure 5:**
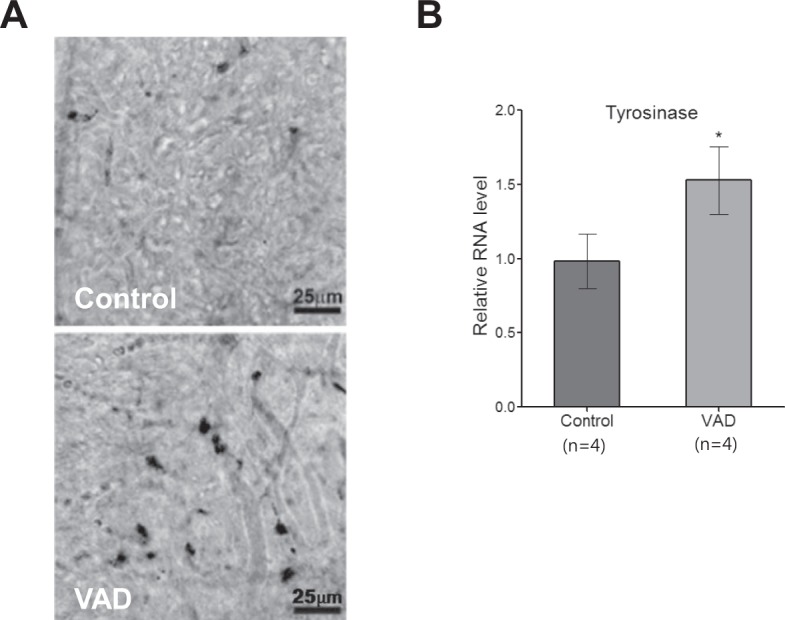
Melanocyte activation in the vitamin A-deficient (VAD) pigmented mice. (**A**) The stria vascularis of the middle cochlear turn was analyzed by confocal microscopy. Bright field images show the melanosome (black pigment) distribution. The population of melanocytes in the stria vascularis of the middle cochlear turn of the VAD mice (n = 4) is greater than that of the control mice (n = 4). Scale bar represents 25 µm. (**B**) The findings of real-time polymerase chain reaction analysis reveal the upregulation of tyrosinase in the cochlea of the VAD pigmented mice. Tissues were harvested from the middle cochlear turn of four C57BL/6 mice from each group. *P < 0.01.

To explain the differences in the patterns of hearing loss between the VAD albino and pigmented mice, we hypothesised that cochlear melanocytes played a protective role against hearing loss under conditions of VAD. Therefore, we compared the proportions of cochlear melanosomes between the VAD and control pigmented mice. Confocal microscopy findings revealed that the population of melanosomes in the stria vascularis in the VAD pigmented mice was greater than in the control pigmented mice (Fig. 5A). Pattern of melanosome expression in all four mice that were assessed was similar to that in the image shown. The expression levels of tyrosinase, an enzyme that induces melanosome formation, in the VAD mice were also 1.53 ± 0.12 times greater than those in the control mice (P < 0.05; Fig. 5B). We further checked the number of cochlear melanocyte (intermediate cell in stria vascularis) by immunostaining of Kir4.1 (KCNJ 10). The number of kir4.1 was not increased in VAD mice (data not shown).

### Changes in hearing threshold after noise exposure in pigmented mice

**Figure 6:**
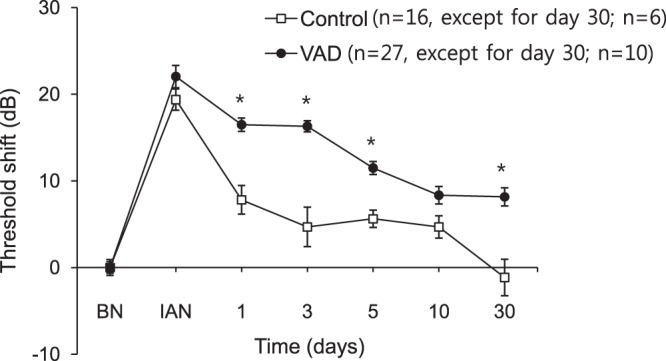
Changes in hearing threshold in response to the clicking sound after noise trauma in the pigmented mice. Significant differences are observed in the restoration of hearing between the vitamin A-deficient (VAD) and control mice 1 day after noise exposure. In the VAD mice, the permanent hearing threshold shift persists even 30 days after noise exposure. The time course of the click ABR threshold shifts after noise exposure (white noise (300–10,000 Hz) at 95-dB SPL for 3 h) in the control (n = 16, n = 6 on day 30) and VAD (n = 27, n = 10 on day 30) mice. *P < 0.05. BN, before noise exposure; IAN, immediately after noise exposure.

The pigmented mice that did not exhibit any changes in hearing threshold despite VAD were subjected to noise trauma in order to induce a transient threshold shift in hearing. Exposure to a 95-dB SPL noise for 3 h induced hearing loss (19.38 ± 1.58-dB shift with the clicking sound) in the control mice. However, the hearing gradually recovered and was eventually completely restored after 30 days. The degree of initial hearing loss in the VAD mice (22.04 ± 1.40-dB shift) was comparable to that in the control mice. However, the VAD mice exhibited delayed recovery of hearing (P < 0.05) and persistence of permanent hearing loss (8.15 ± 1.66-dB shift) even 30 days after noise trauma (P < 0.05; Fig. 6).

**Figure 7:**
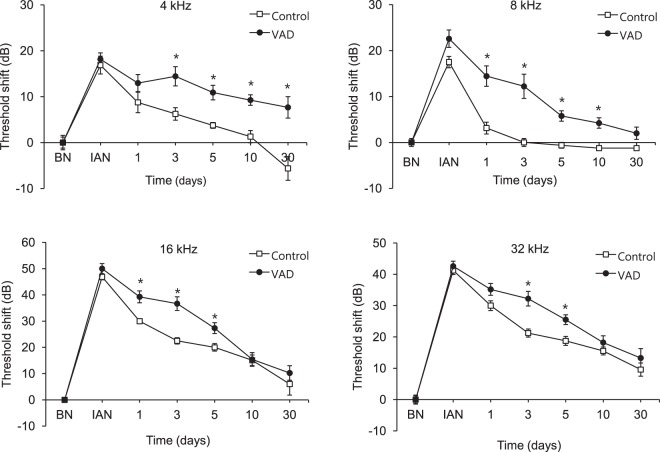
Changes in hearing threshold in the pigmented mice after noise exposure according to frequency. The vitamin A-deficient (VAD) mice exhibit persistence of permanent hearing loss induced by noise exposure at 4 kHz. The time course of the ABR threshold shifts at each frequency in the VAD and control mice after noise exposure (white noise (300–10,000 Hz) at 95-dB SPL for 3 h). *P < 0.05. BN, before noise exposure; IAN, immediately after noise exposure.

In terms of frequency of noise exposure, the control mice exhibited complete recovery of hearing after noise exposure at all frequencies except 32 kHz, which might be indicative of early onset hearing loss in C57BL/6 mice. However, the VAD mice exhibited delayed hearing restoration after noise exposure at 8, 16, and 32 kHz (P < 0.01) and incomplete recovery of hearing after noise exposure at 4 kHz (7.67 ± 2.42-dB shift; P < 0.01; Fig. 7).

## Discussion

In this study, we demonstrated that a complete VAD diet induced severe hearing loss that progressed with age in albino (ICR) mice and that VAD-induced hearing loss resulted in outer hair cell damage, which became more apparent with age. In addition, the older VAD albino mice (16 weeks of age) exhibited neuronal loss in the spiral ganglion. The present findings that VAD mice develop hearing loss that progresses with age contradict the findings of some previous studies involving VAD mouse models, in which no significant hearing loss-induced morphological changes were observed after establishing a VAD diet^9,10^. These inconsistencies might stem from methodological differences between studies when establishing the VAD animal models. In previous studies, the animals were restricted to the VAD diet after birth^7,9^; in contrast, we placed the animals on a VAD diet from 7 to 10 days of gestation. In order to achieve decreased plasma retinol concentrations, a vitamin A-free diet needs to last until the depletion of hepatic reserves. However, since the plasma retinol concentrations remained high enough to prevent the depletion of the hepatic reserves, the previous studies failed to fulfil the requirements for the establishment of complete VAD^7,11^. In the present study, the findings of outer hair cell damage were not closely correlated with the changes in hearing threshold with age because there was no loss of inner hair cells, and the severity of outer hair cell damage was not as high as that observed in hearing loss. These findings suggest that other defects, such as the loss of spiral ganglion neurons, might also be involved in VAD-induced hearing loss.

The mechanism underlying hearing loss in VAD mice remains unclear. One interesting finding of the present study is that pigmented mice did not exhibit hearing loss despite VAD, even though they exhibited significant differences in hearing threshold because of middle-ear effusion at 16 and 18 weeks of age. This difference between the pigmented and albino mouse strains might indicate the role of cochlear melanocytes, which are known to perform antioxidant functions in the inner ear. Melanin is produced by intermediate cells of the stria vascularis, known as strial melanocytes. This pigment, which has free-radical scavenging properties, could play an important role as an antioxidant during oxidative stress-induced cochlear injury in ARHL and NIHL^13,17^. The present findings revealed that VAD results in the upregulation of tyrosinase, which, in turn, results in the increased production of melanocytes to the stria vascularis. Our hypothesis on the mechanism of VAD-induced hearing loss is that VAD induces oxidative stress, which can be protected against by the activation of melanocytes in the pigmented mice. In contrast, because of the lack of cochlear melanocytes, the albino mice cannot exhibit such a protective mechanism, which ultimately results in the development of ARHL.

Although VAD pigmented mice in the present study exhibited normal hearing, there were significant differences in hearing threshold changes after noise exposure between the control and VAD groups of pigmented mice. These results correspond with those of a previous study that found VAD to cause increased sensitivity of the inner ear to noise trauma^7^. The VAD pigmented mice in the present study exhibited poorer restoration of hearing after noise exposure than did the control pigmented mice, with the delay inthe recovery of hearing being especially long at a low frequency (4 kHz) of noise exposure. This might indicate that the otoprotective effect of cochlear melanocytes is insufficient against the double injury of VAD and noise exposure. In other words, upon exposure to noise, the VAD mice were not further protected against oxidative stress because the cochlear melanocytes were already maximally activated for protection against VAD. In contrast, upon noise exposure in the control mice, it was possible for cochlear melanocytes to produce more melanosome, and they were able to protect against the noise-induced oxidative stress.

Even though our data show some evidence that a VAD diet induces hearing loss and that melanocytes may be involved in a protective mechanism for VAD-induced hearing loss, there may be other factors that influence the onset of VAD-induced hearing loss. Notably, VAD diet can cause problems in a variety of organs. For example, VAD induces hyperglycaemia due to the loss of pancreatic beta cell mass, which can cause hearing loss. Differential genetic backgrounds between pigmented and albino mice must be considered when interpreting the different patterns of hearing loss after VAD. As is already known, C57BL/6 mice exhibit a genetic mutation in Cdh23, which causes early onset of age-related hearing loss; additional unknown genetic factors can cause differential phenotypes in VAD.

In summary, VAD induces hearing loss, which can be protected against by cochlear melanocyte activation in pigmented mice. However, albino mice lack this protective mechanism. Moreover, VAD pigmented mice are vulnerable to noise trauma despite the protection afforded by the cochlear melanocytes against hearing loss.

## Methods

### VAD mice

This study included albino ICR mice, which carry a point mutation in exon 1 of the tyrosinase gene 1^18^, and pigmented C57BL/6 mice. Both strains were purchased from Orient Bio Inc. (Seongnam, Korea). Mice of both strains were arbitrarily distributed into two groups: the VAD group, in which the mice were restricted to a chemically defined diet lacking vitamin A (modified AIN-93M without vitamin A; Purina Mills Inc., St Louis, MO, USA), and the control group, in which the mice were maintained on a controlled diet containing 15 IU/g of retinol in the form of retinyl-palmitate (Picolab Rodent Diet 5053; LabDiet, Brentwood, Missouri, USA). These diet regimens were implemented from 7 to 10 days of gestation. Mice in both the groups had adlibitum access to food. The pups were weaned at 3 weeks of age and maintained on the same diet until 20 weeks of age, before being processed for histological analysis. Because of the differences in the age- and noise-related hearing phenotypes between male and female mice, we used only male mice in all the experiments.

This study included 60 ICR (control, 30; VAD, 30) and 80 C57BL/6 (control, 40; VAD, 40) mice. The mice were evaluated for body weight and general morbidity every week until 20 weeks of age. All methods were performed in accordance with the relevant guidelines and regulations. The study protocols were approved by the Institutional Animal Ethics Committee of Yonsei University (permit no. 2015-0018).

### Measurement of serum retinol concentration

Serum retinol concentrations were measured at 4, 8, and 20 weeks of age. The mice were anaesthetised via an intraperitoneal injection of tiletamine/zolazepam (30 mg/kg, Zoletil 50; Virbac S.A., Carros, France) and xylazine hydrochloride (10 mg/kg, Rompun; Bayer Healthcare Korea, Seoul, Korea). Whole blood was collected from the right ventricle of the heart, which was exposed after thoracotomy. Serum retinol concentrations were measured using high-performance liquid chromatography (HPLC; HP1100 series; Hewlett Packard Co., Waldbronn, Germany). All collection and HPLC procedures were performed under N_2_ and reduced light in order to prevent the oxidation of compounds. The lower limit for the detection for serum retinol was 0.03 μmol/L.

### Noise exposure

Noise exposure and evaluation of auditory brainstem response (ABR) were performed in a foam-lined, single-walled, soundproof room (TCA-500S; SONTEK, Gyeonggi-do, Korea). The noise exposure apparatus was specially designed to containan acrylic frame (53 × 35 × 53 × 2 cm; Seoul, Korea) with a speaker (DH1A; Electro-Voice, Burnsville, MN, USA) at the top. White noise (300–10,000 Hz), generated using a computer and an amplifier (R150-plus; Inter M, Seoul, Korea), was delivered through the speaker.

Eight-week-old C57BL/6 (VAD or control group) mice were exposed to a 95-dB peak equivalent sound pressure level (SPL) white noise for 3 h in order to establish transient threshold shift models. To avoid inadequate noise exposure caused by the mice gathering together and hiding their heads during noise stimulation, a specially designed pie-shaped wire cage with eight separated compartments was employed, as described in a previous study^19^. Each separated compartment housed a single mouse.

### Measurement of hearing levels

Hearing levels were determined in each mouse by measuring the ABR thresholds using an auditory-evoked potential workstation and the BioSig software (Tucker-Davis Technologies, Alachua, FL, USA). The output from the speakers was calibrated by using a PCB 377C10 microphone (PCB Piezotronics, Inc. New York, NY, USA) and was found to be within ±4 dB for the frequency range tested. Mice were anaesthetised as described for the collection of whole blood, following which, each ear was stimulated with an ear probe sealed inside the ear canal. Body temperature of the mice was maintained at 38 °C with an isothermic heating water-pad. The intensity of clicking sounds was decreased from 80 dB SPL to 10 dB SPL in 5-dB decrements, while that of the tone pip (10 ms; 11/s) was decreased from 90 dB SPL to 0 dB SPL in 10-dB decrements. The average values of ABR were calculated and the hearing threshold was defined as the lowest recognizable ABR response until wave I of the ABR could no longer be visually discerned. In order to evaluate the changes in hearing threshold with age, ABR was measured every 2 weeks starting from 4 weeks of age. For evaluation of NIHL, ABR was measured before the noise trauma to confirm normal hearing function and immediately after noise exposure on days 1, 3, 5, 10, and 30 in order to determine the magnitude of the threshold shift. Hearing thresholds were recorded as mean values ± standard errors of the mean (SEM).

### Haematoxylin–eosin and phalloidin staining

At 8 or 16 weeks of age, after ABR measurements, the mice (C57BL/6, 16; ICR, 16) were sacrificed using CO_2_ gas, and both cochleae were removed from each mouse. The stapes were excised, and a small hole was made in the cochlear apex by using a fine pick. A fixative (4% formalin and 1% glutaraldehyde in 0.1 M sodium phosphate buffer) was infused through the hole, and the cochlea was immersed in the fixative for 48 hat 4 °C and later preserved in 10% ethylenediaminetetraacetic acid (EDTA) in phosphate-buffered saline (PBS) for 3 days. Half of the excised cochleae were then dehydrated and embedded in paraffin. The paraffin blocks were sectioned into 5-μm slices along the mid-modiolar axis. The sections were observed under a microscope after haematoxylin–eosin staining. The remaining cochleae were washed in PBS for fluorescence staining. The outer bony wall of each cochlea and the tectorial membrane were gently removed, and the organ of Corti was harvested using a fine forceps, beginning from the apex. The structure was then stained with a fluorescein isothiocyanate-conjugated phalloidin probe (Invitrogen, Carlsbad, CA, USA), washed, and examined at a high magnification (600×) under fluorescence and confocal microscopes to evaluate the damaged hair cells. However, the basal region could not be completely examined.

### Scanning electron microscopy

For the scanning electron microscopic study, six ICR mice were sacrificed at 8 or 16 weeks of age. The cochleae were immediately isolated from these mice and perfused carefully through the round window by using a fixative containing 2.5% glutaraldehyde and 2% paraformaldehyde in 0.1 M sodium cacodylate buffer (pH 7.4), after a hole was made at the top of the cochlea. The perfused cochleae were immersed in the same fixation mixture overnight at 4 °C. The lateral wall, Reissner’s membrane, and the tectorial membrane were excised under a dissection microscope and fixed overnight at 4 °C in 0.1 M sodium cacodylate buffer (pH 7.4) containing 2.5% glutaraldehyde and 3.5% sucrose. After fixation, the dissected specimens were rinsed three times for 20 min at 4 °C with 0.1 M sodium cacodylate buffer. They were then subjected to post-fixation by using the osmium tetroxide (OsO_4_)-thiocarbohydrazide method developed by Hunter-Duvar^20^. Briefly, the specimens were immersed in 0.5% OsO_4_ for 1 h at 4 °C and then placed in saturated thiocarbohydrazide for 20 min at room temperature. They were then dehydrated using a graded series of ethanol solutions, dried with a critical point drier (HCP-2; Hitachi, Tokyo, Japan), affixed on a stub, and coated with platinum by using a sputter coater (E1030; Hitachi). The coated specimens were mounted on a stub holder and viewed under a cold-field emission scanning electron microscope (S-4300; Hitachi) operated at 15 kV.

### Real-time polymerase chain reaction for tyrosinase

C57BL/6 mice (VAD diet, 4; normal diet, 4) were sacrificed at 8 weeks of age, and their inner ear was dissected. Total cellular RNA was extracted from the whole inner ear by using TRIzol reagent (Invitrogen, Gaithersburg, MD, USA) and then treated with amplification-grade deoxyribonuclease I (DNase I; Bio-Rad Laboratories, Hercules, CA, USA) before cDNA synthesis. The first cDNA was synthesised using a first cDNA synthesis kit (Invitrogen, Carlsbad, CA, USA) according to the manufacturer’s instructions. For quantification of gene expression, fluorescence real-time polymerase chain reaction (ABI PRISM 7700; PE Applied Biosystems) was performed according to the manufacturer’s instructions by using the double-stranded DNA dye SYBR Green (Perkin-Elmer, Boston, MA, USA). Target gene transcripts were detected using primer pairs for tyrosinase (sense, 5′-GCCTGTGCCTCCTCTAAG-3′; antisense, 5′-TTCTAATCAAGACTCGCTTCTC-3′) and mouse glyceraldehyde 3-phosphate dehydrogenase (GAPDH; control; sense, 5′-TGTGTCCGTCGTGGATCTGA-3′; antisense, 5′-CCTGCTTCACCACCTTCTTGAT-3′). All samples were analysed in duplicate, and target gene expression was normalised to the level of GAPDH expression in each sample. The normalised data were used to quantify the relative RNA levels by ΔCt analysis.

### Analysis of pigmentation

Mice were sacrificed by decapitation and then hemi-sectioned. After removing the brain, the entire inner-ear tissues, including the otic capsule, were dissected and fixed in 4% paraformaldehyde overnight at 4 °C. The oval and round windows were opened for better penetration of the fixative. The specimens were then decalcified in 0.2 M EDTA/diethyl pyrocarbonate-PBS at 4 °C for 2 days, followed by washing with PBS. The lateral wall, including the stria vascularis of the inner ear, was dissected and mounted with 100% glycerol. Pigmentation in the stria vascularis was observed using bright-field laser scanning images acquired using AXIO Imager M2 (Carl Zeiss, Jena, Germany).

### Statistical analysis

All values were presented as mean ± SEM. Serum retinol concentrations were logarithmically transformed to improve normality and to compensate for unequal variance. They were analysed using two-way analysis of variance (ANOVA) followed by post-hoc Bonferroni tests (Supplementary Table 1). Comparison of body weight and hearing parameters among mice categorised on the basis of age and diet was also performed using two-way ANOVA. Post-hoc analyses for comparing the VAD group with the other groups were performed using the Bonferroni test. In addition, independent sample t-tests were applied to determine significant differences in auditory threshold between the different time points in the NIHL model (Supplementary Table 1). Statistical analysis was performed using the SAS software (version 9.2, SAS Institute, Cary, NC, USA), and significance was assumed at P < 0.05.

## Electronic supplementary material

Supplementary Table 1

## Data Availability

The data are available from the corresponding author on reasonable request.
