# Supplementary material for: Progressive hearing loss in vitamin A-deficient mice which may be protected by the activation of cochlear melanocyte
Source: Sci Rep. 2018 Nov 6;8:16415. doi: 10.1038/s41598-018-34653-8 (PMC6219529; doi:10.1038/s41598-018-34653-8)
Supplement: Supplementary file 1 — Supplementary Table 1 [file 41598_2018_34653_MOESM1_ESM.doc]

**Supplementary information**

**Progressive hearing loss in vitamin A-deficient mice which may be protected by the activation of cochlear melanocyte.**

Mia Gi^a#^, Dae Bo Shim^b#^, Ling Wu^a^, Jinwoong Bok^e^, Mee Hyun Song^c^, Jae Young Choi^a,d,f,g,*^

^a^Brain Korea 21 PLUS Project for Medical Science, Yonsei University College of Medicine, Seoul, South Korea

^b^Department of Otorhinolaryngology, Myongji Hospital, Hanyang University College of Medicine, Goyang, South Korea

^c^Department of Otorhinolaryngology, Myongji Hospital, Hanyang University Medical Center, Goyang, South Korea

^d^Department of Otorhinolaryngology, Yonsei University College of Medicine, Seoul, South Korea

^e^Department of Anatomy, Brain Korea 21 PLUS Project for Medical Science, Yonsei University College of Medicine, Seoul, South Korea

^f^Research Center for Natural Human Defense System, Yonsei University College of Medicine, Seoul, South Korea

^g^The Airway Mucus Institute, Yonsei University College of Medicine, Seoul, South Korea

**Supplementary Table 1. Statistical analysis**

Statistical analyses were performed by using a two-way ANOVA, except for the noise-induced hearing loss study where statistical analyses were performed by using a t-test.

ABR, auditory brainstem response; VAD, vitamin A deficiency.
